# Supplementary material for: Modeling glioblastoma heterogeneity as a dynamic network of cell states
Source: Mol Syst Biol. 2021 Sep 16;17(9):e10105. doi: 10.15252/msb.202010105 (PMC8444284; doi:10.15252/msb.202010105)
Supplement: Supplementary file 6 — Source Data for Figure 5 [file MSB-17-e10105-s004.zip › Figure5A_sourcedata/GSEA_3071/nosigdb_stateII.GseaPreranked.1622795151171/RICHARDS_ZHONG_NATURE_2018_OPC_UPREG.html]

Details for gene set RICHARDS\_ZHONG\_NATURE\_2018\_OPC\_UPREG[GSEA]

|  || Dataset | state23071 |
| Phenotype | NoPhenotypeAvailable |
| Upregulated in class | na\_neg |
| GeneSet | RICHARDS\_ZHONG\_NATURE\_2018\_OPC\_UPREG |
| Enrichment Score (ES) | -0.7332294 |
| Normalized Enrichment Score (NES) | -3.4816859 |
| Nominal p-value | 0.0 |
| FDR q-value | 0.0 |
| FWER p-Value | 0.0 |
Table: GSEA Results Summary

  

Fig 1: Enrichment plot: RICHARDS\_ZHONG\_NATURE\_2018\_OPC\_UPREG      
 Profile of the Running ES Score & Positions of GeneSet Members on the Rank Ordered List

  

| PROBE | GENE SYMBOL | GENE\_TITLE | RANK IN GENE LIST | RANK METRIC SCORE | RUNNING ES | CORE ENRICHMENT || 1 | ALCAM |  |  | 288 | 0.771 | -0.0557 | No |
| 2 | EPB41L2 |  |  | 623 | 0.569 | -0.1268 | No |
| 3 | PSAT1 |  |  | 1505 | 0.340 | -0.3375 | No |
| 4 | ARL4A |  |  | 2167 | 0.255 | -0.4956 | No |
| 5 | ANXA5 |  |  | 2187 | 0.253 | -0.4953 | No |
| 6 | OLIG2 |  |  | 2453 | -0.278 | -0.5551 | No |
| 7 | CNP |  |  | 2457 | -0.278 | -0.5503 | No |
| 8 | GATM |  |  | 2501 | -0.285 | -0.5552 | No |
| 9 | MMP2 |  |  | 2762 | -0.328 | -0.6129 | No |
| 10 | PDE4B |  |  | 2788 | -0.333 | -0.6124 | No |
| 11 | TSC22D4 |  |  | 2847 | -0.344 | -0.6198 | No |
| 12 | SLITRK2 |  |  | 2970 | -0.368 | -0.6426 | No |
| 13 | ANGPTL2 |  |  | 3019 | -0.378 | -0.6469 | No |
| 14 | PLEKHB1 |  |  | 3131 | -0.409 | -0.6661 | No |
| 15 | SPRY4 |  |  | 3201 | -0.429 | -0.6746 | No |
| 16 | PON2 |  |  | 3216 | -0.432 | -0.6694 | No |
| 17 | RFTN2 |  |  | 3427 | -0.511 | -0.7110 | No |
| 18 | ITM2C |  |  | 3518 | -0.552 | -0.7222 | Yes |
| 19 | OLIG1 |  |  | 3551 | -0.571 | -0.7187 | Yes |
| 20 | CD9 |  |  | 3567 | -0.581 | -0.7107 | Yes |
| 21 | DBI |  |  | 3597 | -0.601 | -0.7059 | Yes |
| 22 | SPATA6 |  |  | 3606 | -0.608 | -0.6957 | Yes |
| 23 | GNG7 |  |  | 3746 | -0.724 | -0.7155 | Yes |
| 24 | RAB31 |  |  | 3757 | -0.737 | -0.7033 | Yes |
| 25 | SCD5 |  |  | 3765 | -0.751 | -0.6900 | Yes |
| 26 | PLAT |  |  | 3766 | -0.752 | -0.6749 | Yes |
| 27 | TTYH1 |  |  | 3807 | -0.795 | -0.6689 | Yes |
| 28 | SOX8 |  |  | 3809 | -0.797 | -0.6532 | Yes |
| 29 | DUSP6 |  |  | 3899 | -0.946 | -0.6563 | Yes |
| 30 | SEMA5A |  |  | 3921 | -1.006 | -0.6414 | Yes |
| 31 | BCHE |  |  | 3926 | -1.012 | -0.6221 | Yes |
| 32 | ETV1 |  |  | 3934 | -1.026 | -0.6033 | Yes |
| 33 | TRIB2 |  |  | 3944 | -1.041 | -0.5847 | Yes |
| 34 | SOX6 |  |  | 3956 | -1.081 | -0.5658 | Yes |
| 35 | ITGB8 |  |  | 3962 | -1.093 | -0.5452 | Yes |
| 36 | ETV5 |  |  | 3971 | -1.116 | -0.5248 | Yes |
| 37 | CD82 |  |  | 3973 | -1.119 | -0.5027 | Yes |
| 38 | NOVA1 |  |  | 4008 | -1.312 | -0.4849 | Yes |
| 39 | LSAMP |  |  | 4028 | -1.442 | -0.4607 | Yes |
| 40 | SLC35F1 |  |  | 4035 | -1.515 | -0.4319 | Yes |
| 41 | SPRY1 |  |  | 4044 | -1.573 | -0.4024 | Yes |
| 42 | LIMA1 |  |  | 4056 | -1.716 | -0.3708 | Yes |
| 43 | SCRG1 |  |  | 4070 | -2.021 | -0.3336 | Yes |
| 44 | PTN |  |  | 4077 | -2.187 | -0.2913 | Yes |
| 45 | S100B |  |  | 4081 | -2.383 | -0.2444 | Yes |
| 46 | PMP2 |  |  | 4082 | -2.398 | -0.1965 | Yes |
| 47 | GPM6B |  |  | 4083 | -2.435 | -0.1478 | Yes |
| 48 | PDGFRA |  |  | 4096 | -3.521 | -0.0803 | Yes |
| 49 | PTPRZ1 |  |  | 4097 | -4.027 | 0.0002 | Yes |
Table: GSEA details [plain text format]

  

Fig 2: RICHARDS\_ZHONG\_NATURE\_2018\_OPC\_UPREG: Random ES distribution      
 Gene set null distribution of ES for **RICHARDS\_ZHONG\_NATURE\_2018\_OPC\_UPREG**
